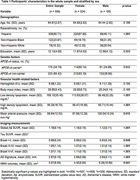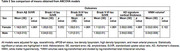# Sex differences in imaging biomarkers of Alzheimer’s pathology, neurodegeneration, and vascular injury in a diverse cohort of late middle‐aged adults

**DOI:** 10.1002/alz70861_108314

**Published:** 2025-12-23

**Authors:** Muge Akinci, Froogh Aziz, Priya Palta, Diana S. Guzmán, Jeanne Teresi, Adam Brickman, Patrick J. Lao, Jose A. Luchsinger

**Affiliations:** ^1^ Department of Medicine, College of Physicians and Surgeons, Columbia University Irving Medical Center, New York, NY USA; ^2^ Department of Epidemiology, Joseph P. Mailman School of Public Health, Columbia University Irving Medical Center, New York, NY USA; ^3^ University of North Carolina at Chapel Hill, Chapel Hill, NC USA; ^4^ Taub Institute for Research on Alzheimer’s Disease and the Aging Brain, Columbia University Irving Medical Center, New York, NY USA; ^5^ Columbia University Stroud Center, Department of Medicine and New York State Psychiatric Institute, New York, NY USA; ^6^ Gertrude H. Sergievsky Center, Columbia University Irving Medical Center, New York, NY USA; ^7^ Department of Neurology, Vagelos College of Physicians and Surgeons, Columbia University, New York, NY USA

## Abstract

**Background:**

Whether females have a higher risk of Alzheimer’s disease (AD)‐related pathologies than males remains unclear. We examined sex differences in amyloidosis, tau pathology, neurodegeneration, and vascular injury in a multi‐ethnic cohort of late middle‐aged adults. We further explored potential moderators of these differences.

**Methods:**

This cross‐sectional cohort study included 509 cognitively unimpaired adults (mean age: 64.7±2.87, 63.6% female) from Northern Manhattan, New York City. Of all participants, 60.7% were self‐identified as Hispanic, 23.9% as non‐Hispanic Black, and 15.3% as non‐Hispanic White (Table 1). Participants underwent amyloid positron emission tomography (PET) imaging with 18F‐Florbetaben, tau PET imaging with 18F‐MK‐6240 (*n* =358), and structural magnetic resonance imaging (MRI). MRI‐derived cortical thickness in AD signature regions (limbic and heteromodal association areas) and white matter hyperintensity (WMH) volumes were used as markers of neurodegeneration and vascular injury, respectively. We performed analyses of covariance to examine sex differences in global amyloid burden, tau burden across early, middle, and late accumulation areas (Braak‐stage I/II, Braak‐stage III/IV, and Braak‐stage V/VI, respectively), AD signature cortical thickness, and WMH volumes. Covariates included demographics, *APOE‐*e4 status, and vascular health‐related factors (Table 1). We then explored effect modification by age, *APOE‐*e4 status, and race/ethnicity by introducing sex‐by‐age, sex‐by‐race/ethnicity, and sex‐by‐*APOE‐*e4 status interaction terms into the models.

**Results:**

Compared to males, females had significantly higher brain amyloid burden and tau burden in the Braak III/IV and Braak V/VI regions, and had greater cortical thickness in AD signature areas (Table 2). We did not find statistically significant sex differences in Braak I/II tau burden and WMH volumes. Further, we observed a significant sex‐by‐*APOE‐*e4 interaction on tau burden such that among *APOE‐*e4 carriers, females showed greater tau burden in the Braak I/II (*p*=0.007), Braak III/IV (*p*=0.008), and Braak V/VI (*p*=0.016) regions compared to males. Sex differences in the outcomes did not vary by age or race/ethnicity.

**Conclusions:**

In a late middle‐aged, diverse urban cohort, females had higher amyloid and tau burden, as well as greater cortical thickness in AD‐vulnerable regions, compared with males. Greater tau burden in females appears be more pronounced beyond early tau accumulation areas and among *APOE‐*e4 carriers.